# Perspectives of traditional herbal medicines in treating retinitis pigmentosa

**DOI:** 10.3389/fmed.2024.1468230

**Published:** 2024-12-06

**Authors:** Shihui Liu, Toshihiko Matsuo, Chie Matsuo, Takumi Abe, Jinghua Chen, Chi Sun, Qing Zhao

**Affiliations:** ^1^Graduate School of Interdisciplinary Science and Engineering in Health Systems, Okayama University, Okayama, Japan; ^2^Department of Ophthalmology, Shanghai General Hospital (Shanghai First People's Hospital), Shanghai Jiao Tong University, National Clinical Research Center for Eye Diseases, Shanghai Key Laboratory of Ocular Fundus Diseases, Shanghai Engineering Center for Visual Science and Photomedicine, Shanghai Engineering Center for Precise Diagnosis and Treatment of Eye Diseases, Shanghai, China; ^3^Department of Ophthalmology, Okayama University Hospital, Okayama, Japan; ^4^Graduate School of Medicine, Dentistry and Pharmaceutical Sciences, Okayama University, Okayama, Japan; ^5^Department of Ophthalmology, University of Florida, College of Medicine, Gainesville, FL, United States; ^6^Department of Ophthalmology and Visual Sciences, Washington University in St. Louis, St. Louis, MO, United States; ^7^National Key Laboratory of Plant Molecular Genetics, CAS Center for Excellence in Molecular Plant Sciences, Shanghai Institute of Plant Physiology and Ecology, Chinese Academy of Sciences, Shanghai, China; ^8^Shanghai Key Laboratory of Plant Functional Genomics and Resources, Shanghai Chenshan Botanical Garden, Shanghai Chenshan Plant Science Research Center, Chinese Academy of Sciences, Shanghai, China

**Keywords:** retinitis pigmentosa, ophthalmology, botany, pharmacology, medical history, compound, drug discovery, degenerative diseases

## Abstract

Medicinal plants, also known as herbs, have been discovered and utilized in traditional medical practice since prehistoric times. Medicinal plants have been proven rich in thousands of natural products that hold great potential for the development of new drugs. Previously, we reviewed the types of Chinese traditional medicines that a Tang Dynasty monk Jianzhen (Japanese: Ganjin) brought to Japan from China in 742. This article aims to review the origin of Kampo (Japanese traditional medicine), and to present the overview of neurodegenerative diseases and retinitis pigmentosa as well as medicinal plants in some depth. Through the study of medical history of the origin of Kampo, we found that herbs medicines contain many neuroprotective ingredients. It provides us a new perspective on extracting neuroprotective components from herbs medicines to treat neurodegenerative diseases. Retinitis pigmentosa (one of the ophthalmic neurodegenerative diseases) is an incurable blinding disease and has become a popular research direction in global ophthalmology. To date, treatments for retinitis pigmentosa are very limited worldwide. Therefore, we intend to integrate the knowledge and skills from different disciplines, such as medical science, pharmaceutical science and plant science, to take a new therapeutic approach to treat neurodegenerative diseases. In the future, we will use specific active ingredients extracted from medicinal plants to treat retinitis pigmentosa. By exploring the potent bioactive ingredients present in medicinal plants, a valuable opportunity will be offered to uncover novel approaches for the development of drugs which target for retinitis pigmentosa.

## 1 Introduction

Plants have long served as the primary source of medicinal compounds since the advent of humanity. Plants harbor a vast array of compounds, and these various compounds have tremendous potential in the development of future medicines. Here, we explore the realm of herbal medicine and plant science in relation to retinal neurodegenerative diseases. There has been limited research focused on extracting active ingredients from plants specifically for the treatment of retinitis pigmentosa. Therefore, a multidisciplinary approach is necessary to uncover effective solutions for these conditions. Collaborations across various disciplines such as plant science (1, 2), pharmaceutical science (3, 4), and medical science (5–10) can yield synergistic outcomes and contribute to the development of more sophisticated treatments for retinitis pigmentosa.

## 2 Overview of the neurodegenerative diseases

Neurodegenerative diseases (NDDs) are characterized by the gradual loss of neurons and/or their myelin sheath, leading to functional deterioration over time. Within the field of neurology, prominent neurodegenerative diseases include Alzheimer disease (AD), Parkinson disease (PD), amyotrophic lateral sclerosis (ALS), and more. Similarly, in the realm of ophthalmology, neurodegenerative diseases encompass retinitis pigmentosa, age-related macular degeneration, glaucoma, and other related conditions.

Neurodegenerative diseases are driven by various shared pathogenic mechanisms, including: (1) abnormal protein dynamics characterized by protein misfolding and aggregation; (2) oxidative stress resulting from the formation of reactive oxygen species and free radicals; (3) dysfunction of neurotrophic factors; (4) mitochondrial dysfunction; (5) neuroimmune inflammation; (6) failure of neuronal Golgi apparatus; (7) disruption of cell/axonal transport; and (8) altered cell signaling. The convergence of these diverse pathogenic factors ultimately leads to multifaceted neuronal cell death (11–14). Effective therapeutic strategies aim to tackle these pathogenic mechanisms to halt or delay disease progression.

Developing therapeutic strategies for neurodegenerative diseases has profoundly drawn much attention from the public and medical field. On December 23, 2021, President Biden signed the Accelerated Access to Critical Treatments (ACT) for amyotrophic lateral sclerosis (ALS) Act (ACT for ALS) into law as Public Law 117–79. This legislation mandates the Department of Health and Human Services (HHS) to collaborate with the Food and Drug Administration (FDA) and the National Institutes of Health (NIH) in establishing public-private partnerships focused on rare neurodegenerative diseases. These partnerships will be facilitated through collaborative agreements or contracts, with the goal of enhancing our understanding of these diseases and expediting the development of treatments for ALS and other related conditions (whitehouse.org). The FDA has devised a comprehensive five-year plan to promote innovation, has streamlined processes, and accelerated the development of drugs specifically tailored for treating rare neurodegenerative diseases, including ALS (fda.org). Consequently, scientists around the world are actively engaged in research, development, and advancement of drugs aimed at addressing the treatment needs of individuals with rare neurodegenerative diseases, steering us toward a brighter future in this field.

## 3 Overview of the origin of Kampo (Japanese traditional medicine)

Monk Jianzhen, also known as Ganjin in Japanese, is renowned in Japan for his teachings on Buddhist precepts (Vinaya). Additionally, he played a crucial role in introducing Chinese herbal medicines to Japan. He not only cultivated medicinal plants within the country but also instructed his disciples on the identification and prescription of medicinal materials. Unfortunately, he lost his eyesight upon arriving in Nara, Japan (15). This event can be seen as the planting of a seed in the land, which subsequently led to numerous developments, including the field of pharmacognosy, the formulation of various Kampo medicines, and the treatment of countless people.

Jianzhen had five unsuccessful attempts to travel to Japan which finally led to achieving success on his sixth try. He lost his eyesight, when he arrived in Japan. During that era, these was no advanced medical technology for diagnosing the cause of blindness. Numerous sight-threatening conditions, such as cataract, glaucoma, age-related macular degeneration (AMD), diabetic retinopathy, as well as ophthalmological neurodegenerative disorders like retinitis pigmentosa (RP), remained undiagnosed and untreated. In contrast, contemporary medical advancements enable the diagnosis of these eye diseases and provide treatments to improve vision. Among the prominent areas of research in ophthalmology, retinitis pigmentosa stands out. Regrettably, most patients with this disease have no choice but to endure progressive vision loss.

## 4 Overview of the retinitis pigmentosa

Inherited retinal diseases (IRDs) are a group of inherited eye disorders that alter the structure and function of the retina, leading to vision loss and sometimes blindness. Eye is highly compartmentalized and shows the immune privilege during retinal degeneration (16, 17) which becomes an ideal tissue to evaluate genetic and pharmacological therapies. There are many types of IRDs, such as Retinitis pigmentosa (RP), Rod dystrophy or rod-cone dystrophy, Usher syndrome (USH), Bietti crystalline dystrophy (BCD), Alport syndrome, Leber congenital amaurosis (LCA) or early onset retinal dystrophy (EORD), Cone dystrophy, Cone-rod dystrophy (CORD), Macula dystrophy, Stargardt’s disease, Best disease, X-linked retinoschisis (XLRS). Most of IRDs affect the photoreceptor cells, which reduces or prevents the retina’s response to light and causes vision loss. Photoreceptors are light-sensing neurons that capture and convert light photons to electrical signals at the specialized primary cilia called outer segments (18). Two classes of photoreceptors, rods and cones, are found in the outer nuclear layer (ONL) of the retina that is located at the back of an eye. Rods are more sensitive in the condition of dim light, whereas cones are more sensitive in daylight and responsible for color vision (19, 20). In addition, cones are concentrated in the central area (i.e., macula) of a human retina (21). Cones localize in the center of the retina at the fovea. There are approximately 6 million cones and more than 100 million rods in a human retina (22). The most common IRD is retinitis pigmentosa (RP). RP is characterized by a progressive loss of rods followed by the concomitant loss of cones (23). The disease syndrome is commonly exemplified by night blindness or nyctalopia at early stages, accompanied by the deterioration of abnormal rod-driven electroretinogram. As RP progresses to later stages, gradual ocular fundus changes become noticeable, primarily encompassing a triad of optic disk pallor, attenuation of retinal blood vessels, and the dispersion of bone spicule-like pigments (24). Extensive loss of photoreceptors eventually leads to tunnel vision or blindness. RP inheritance can be autosomal dominant, autosomal recessive, or X-linked recessive (23). The advancement of genetic testing has identified the genetic causes of IRDs for approximately two thirds of associated patients (25, 26), including cases of RP. RP-associated genes include rod-function genes such as *RHO*, *ABCA4*, *CNGA1*, and *CNGB1*, and rod-specific transcription factor genes *NRL* and *NR2E3* (27–29) (retnet.org).

RP is currently uncurable for the majority of patients. Gene therapy, particularly gene augmentation/replacement, yields a new hope for these patients. LUXTURNA (voretigene neparvovec-rzyl) is a prescription gene therapy product used for the treatment of patients with recessive mutations in *RPE65* gene (30). Many clinical trials are testing AAV-mediated gene therapies for various forms of RP (31, 32). Unfortunately, gene therapy does not cover all forms and onsets of RPs. Some of the advantages of herbal medicines and extracted compounds in treatment for IRDs include: (1) Genetic testing cannot detect all RP-associated genes or compound heterozygosity. Hence, gene therapy may not be applicable to patients with unknown genetic causes. These patients would opt for other forms of medication to delay the disease progression. Pharmacological interventions are gaining great popularity for early-stage RP (33–35). Many preclinical tests involve the therapeutic applications of antioxidants and other natural products to RP-related animal and/cell models (details are discussed in the following section). (2) Gene therapy usually targets early-onset IRDs. There is no clinical trial of genetic interventions for mid- and late-onset RP. In addition, the clinical diagnosis of mid- and late-onset neurodegenerative diseases often suffers from the low specificity of individual assessment methods and biomarkers (36). Hence, antioxidants provide the conservative treatment to patients with mid- and late-onset RP. (3) Different forms of RP demonstrate variable onsets and patterns of disease progression (37, 38). The accuracy of forecasts for the disease progression is unavailable. The herbal medicines would prolong the window of opportunity for symptom monitoring and further interventions. (4) The herbal medicines would serve as a general therapeutic strategy, regardless of the specific mutations, aging conditions, and spatial patterns. (5) The herbal medicines would become a valuable second-line therapy to enhance the efficacy of gene therapy, chemical intervention (39–42) and cell transplantation (43).

## 5 Overview of the medicinal plant

### 5.1 The history of medicinal plant use

The history of medicinal plants dates back thousands of years, with various archeological discoveries attesting to their early use. For instance, a cave in South Africa revealed 77,000-year-old beds constructed from the anti-mosquito plant Cryptocarya woodii (44). Dental calculus from Neanderthals dating back 49,000 years (during the Paleolithic Age) contained residues of Asteraceae plants such as *Matricaria chamomilla* (chamomile) and *Achillea millefolium* (common yarrow) (45), that have functions of alleviating toothache. In Xiaoshan, Hangzhou, Zhejiang, an 8,000-year-old tea tree seed was found at the Kuahuqiao site (46), alongside pottery cauldron cooking utensils containing plant residues associated with herbal medicine. Archeologists also discovered a range of medicinal plants, including Ziziphus jujube, Gorgon fruit, and water chestnut, in the ruins of the 5,000-year-old Liangzhu ancient city (47).

**Figure 1:**
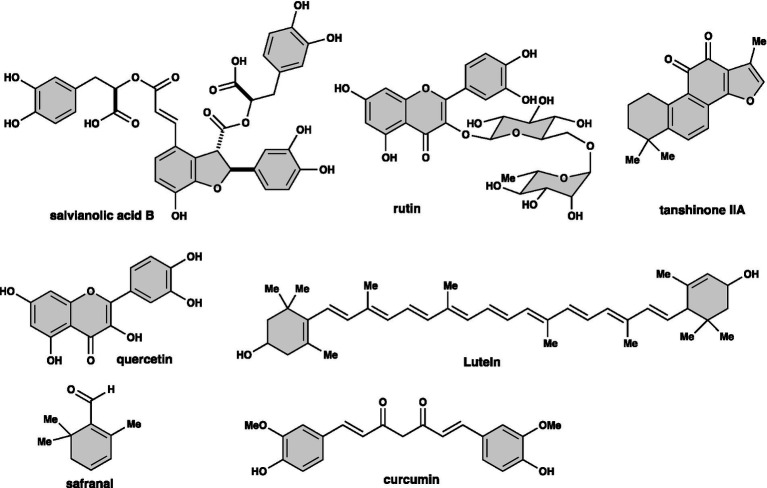
Structures of the main component of some Chinese herbal medicines.

Traditional Chinese medicine (TCM) holds a prominent position with its long-standing history of thousands of years, serving as a global representative of traditional medicine. In the quest for disease treatment and health maintenance, more and more people have turned to natural medicines and green plants (Figure 1). Traditional medicine practices are often passed down through generations orally. Jianzhen’s prescription, for example, has been verbally transmitted for over 1,000 years and has now been compiled into a book by Lei Yutian, a 52nd generation inheritor, making it available to the public (15).

China, specifically, boasts numerous traditional Chinese medicine formulas that demonstrate potent therapeutic effects, although their standardization still requires international recognition. Such traditional medicinal practices can be found worldwide and harbor significant therapeutic potential. However, many of these herbal medicines are at risk of disappearing due to insufficient documentation. Establishing relevant regulations and policies for their protection would elevate the standards of traditional herbal medicines to international levels, ultimately benefiting a greater number of patients.

In TCM, various parts of natural plants, such as roots, stems, and leaves, are utilized as medicinal materials (Figure 1). Each Chinese medicine recipe contains hundreds of known and unknown chemical compounds. Presently, China recognizes over 10,000 types of traditional Chinese medicines, serving as a vast reservoir of organic compounds waiting to be explored. From these organic precursor compounds, diverse drugs for numerous diseases can be developed in the future.

**Figure 2:**
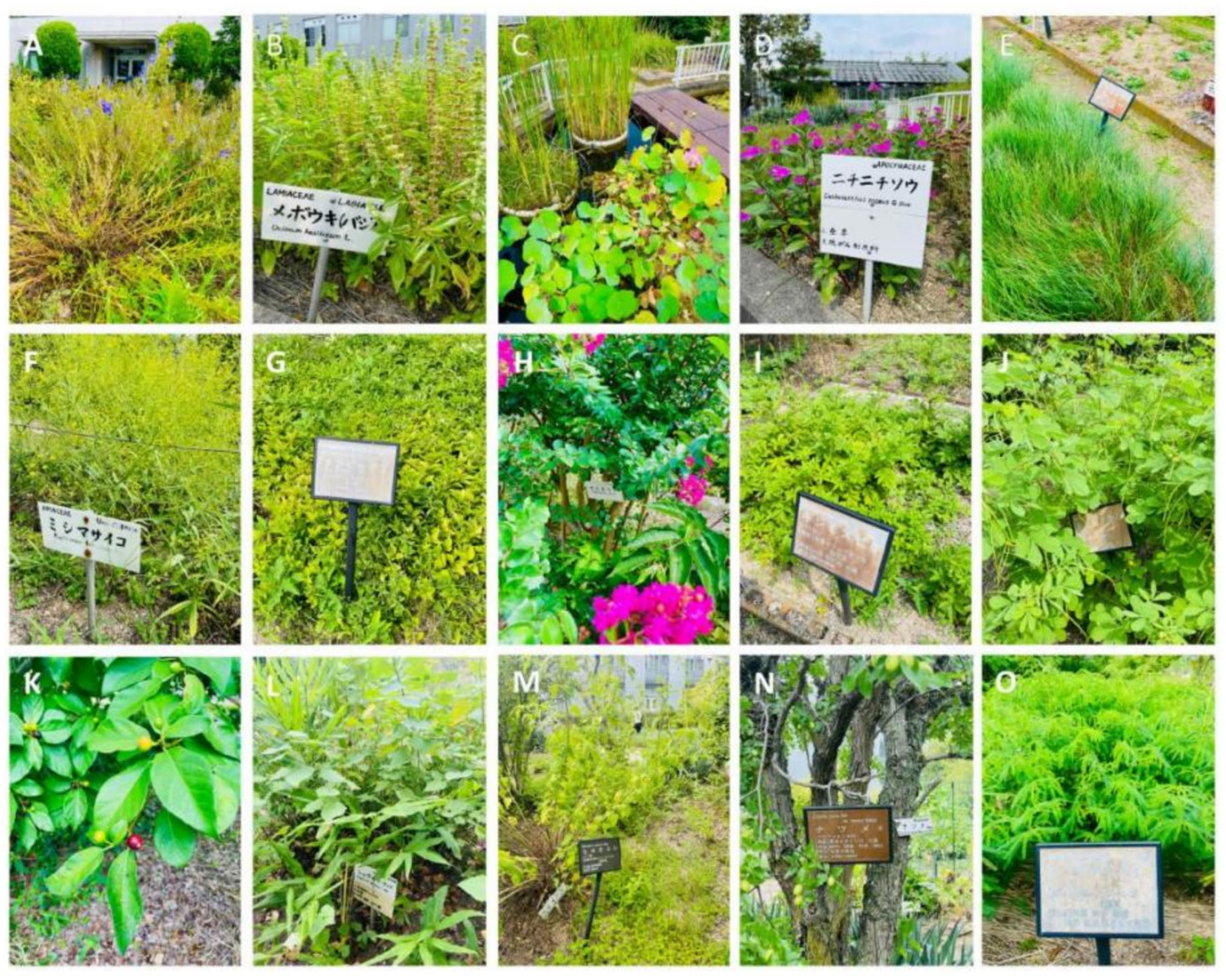
Medicinal plants for Kampo Medicines in Medical Botanical Garden (Okayama University, Japan). **(A)** Scutellaria baicalensis Georgi, **(B)** *Ocimum basilicum* L., **(C)** Typha orientalis C.Presl, *Nymphaea tetragona* georgi, **(D)** *Catharanthus roseus* (L.) G.Don, **(E)** Ephedra intermedia Schrenk & C.A.Mey., **(F)** Bupleurum chinense DC., **(G)** Stemona sessilifolia (Miq.) Miq., **(H)** *Lagerstroemia indica* L., **(I)** Agrimonia pilosa Ledeb., **(J)** *Senna obtusifolia* (L.) H.S.Irwin & Barneby, **(K)** Ficus erecta Thunb., **(L)** Fagopyrum cymosum (Trevir.) Meisn., **(M)** Isodon japonicus (Burm.f.) H.Hara, **(N)** *Ziziphus jujuba* Mill. **(O)** Chamaecrista nomame (Makino) H.Ohashi.

Japan, with its distinct approach, approves Kampo medicines based on governmental assessments of their safety, effectiveness, quality, and manufacturing control. These medicines are prescribed alongside Western medicines under national health insurance and are also available as over-the-counter drugs in pharmacies. The materials used in Kampo medicines are derived from medicinal plants (Figure 2). The plant names were checked with http://mpns.kew.org. It has been discovered through recent research that the origin of the structured and systematic prescriptions in Japanese Kampo medicine can be attributed to Jianzhen, who arrived in Japan during the Nara period in the eighth century. Some herbal medicines brought by Jianzhen are preserved in the Shoso-in of Todaiji Temple, and certain prescriptions are included in the oldest medical book, “Ishin-ho,” compiled by Tanba Yasuyori in 984, albeit with limited details available.

### 5.2 Current market value of medicinal plant compounds

Medicinal plant compounds have a long history, and many early medicines were derived from natural metabolites found in plants. Prominent examples of plant-derived medications include aspirin, quinine, and digoxin. Plants contain a vast array of compounds, including secondary metabolites, which can be broadly categorized into three groups: phenolics, terpenoids, and alkaloids (48). Numerous natural compounds from these plants which now serve as active ingredients in many modern pharmaceuticals. The global pharmaceutical market, valued at around US$1.1 trillion annually, relies on the drugs derived from natural products. Among these sources, plants contribute 25%, microorganisms contribute 13%, and animals contribute 3% (49). The utilization of natural products offers several advantages compared to other sources. These compounds exhibit chemical novelties that can serve as starting points for developing potential drug candidates targeting complex diseases. Moreover, naturally derived ingredients possess chemical diversity, intricate bi- and tri-dimensional structures, and can be efficiently absorbed and metabolized within the body (50). Medicinal plant compounds encompass a variety of active ingredients and secondary metabolites that demonstrate favorable properties, including anti-inflammatory, anti-bacterial, antiviral, anti-cancer, antioxidant, and anti-apoptotic effects. In this article, we provide a review of select medicinal plant components employed in the treatment of retinitis pigmentosa, and we anticipate future research directions and advancements in the application of medicinal plants within this field. The significant role of medicinal plants as invaluable resources for the development of new drugs within the global pharmaceutical industry cannot be overstated (Table 1).

**Table 1:** Representative herbal medicines for the treatment of Retinitis Pigmentosa.

No.	Chinese herbal medicines	Extraction source	Pharmacological effects in the treatment of retinitis pigmentosa	Animal model of retinitis pigmentosa
1	*Lycium barbarum* polysaccharide (LBP)	Goji berry fruit (*Lycium barbarum* L.)	Antioxidant effect	rd1
2	Salvianolic acid B	The root and rhizome of Danshen (*Salvia miltiorrhiza* Bunge)	Antioxidant effect	rd10
3	Tanshinone IIA	The root of Danshen (*Salvia miltiorrhiza* Bunge)	Antioxidant effect	rd10
4	Rutin	Citrus fruits, buckwheat, asparagus, and apples, etc.	Antioxidant effect	rd10
5	Quercetin	Peppers, onions, berries, broccoli and red apples, etc.	Antioxidant effect	rd10
6	Lutein	Spinach, kale, and broccoli, etc.	Antioxidant effect	rd10
7	Safranal	Stigmas of saffron (*Crocus sativus* L.)	Antioxidant effect, antiapoptotic effect	P23H
8	Curcumin	Turmeric ginger (*Zingiber officinale* Roscoe)	Antioxidant effect	P23H

## 6 Phytogenic compounds currently in development to treat retinitis pigmentosa

Herbal medicines currently being developed for the treatment of retinitis pigmentosa include *Lycium barbarum* polysaccharide, salvianolic acid B, tanshinone IIA, rutin, quercetin, lutein, Safranal, curcumin, etc. (Figure 1), which mainly protect retinal nerve cell damage through the antioxidant properties of herbal medicine. Numerous clinical and epidemiological studies have demonstrated the beneficial effects of these plant-derived compounds on ocular diseases (51). More importantly, these compounds have been tested in animal models of retinitis pigmentosa (details are listed below).

Major animal models of retinitis pigmentosa are described as follows. (1) Mice homozygous for the retinal degeneration 1 (*rd1*) mutation have an early-onset severe rod degeneration mainly due to a nonsense mutation in exon 7 of the *Pde6b* gene (52, 53) which causes undetectable PDE6B protein. At postnatal day (P) 10, the rod outer segment shows signs of disruption, the apoptosis of photoreceptor cells increases with a rapid loss of rods by P14 (age of eye opening). Rod degeneration happens in prior to cone degeneration in all regions of the retina. By P21, less than 2% of rods can be found in *rd1/rd1* mice but more than half of cones are still present (54). Expression of rod-enriched genes is downregulated in *rd1/rd1* mice (55, 56). (2) The retinal degeneration 10 (*rd10)* mouse carries a missense mutation (R560C) in exon 13 of the *Pde6b* gene (57), causing a reduced PDE6B protein expression. Photoreceptor loss occurs from the central retina at P16 and spreads to the peripheral retina around P20 in homozygous mutants, for which the peak of apoptosis happens between P18 and P25. ONL is fully degenerated by P60 (58). (3) Autosomal dominant *Rho^P23H^* is the most frequent RP mutation (59). Rod outer segments appear shorter in *Rho^P23H/+^* mouse retinas at P35, and about 50% ONL neurons are lost in mutants at P63 (60). The pathogenic mechanism indicates that a misfolded monomer of P23H opsin induces aggregation of mutant rhodopsin protein with WT counterpart and prevents the formation of rod outer segment.

1. *Lycium barbarum* polysaccharide (LBP). LBPs are a group of water-soluble glycoconjugates and can be extracted from wolfberry or goji berry (*Lycium barbarum L.*) (61, 62). Goji berry is a common herb of traditional Chinese medicine (63). Regular treatment of LBPs benefited the neuronal survival in studies of animal models (64–67). In particular of retinopathies, LBPs could preserve neurons (ex: rod bipolar cells and amacrine cells) of inner nuclear layer in the model of retinal ischaemia (68), and protect retinal ganglion cells from CoCl_2_-induced apoptosis (69). Notably, LBPs delayed the ensuing degeneration of retinal ganglion cells and cone photoreceptors in *rd1* mice (70), which matched results of a placebo-controlled intervention trial with 12-month LBP oral administration (71). Since the therapeutic window for rods appeared longer in *rd10* mice, LBP treatment reduced photoreceptor apoptosis via inhibition of through inhibition of NF-κB and HIF-1α pathways and improved scotopic and photopic electroretinogram responses. In addition, LBP treatment could inhibit the activation of microglia in *rd10* retinas (72). However, polysaccharides have limited solubility in conventional solvent system, leading to difficulties in effective extraction and co-delivery with other compounds (73).
2. Salvianolic acid B (Sal B). Sal B is a 1-benzofuran derived from the root and rhizome of the plant species Danshen (*Salvia miltiorrhiza* Bunge), which has been commonly used in traditional Chinese medicine for various therapeutic purposes (74, 75). Sal B is well known for its anti-apoptotic and anti-inflammatory properties in treating neurodegenerative diseases via associated pathways of PI3K/Akt (76, 77), AMPK (78, 79), SIRT1 (78, 80) etc. Furthermore, studies have explored the therapeutic effects of Sal B in RPE and lens diseases, indicating its anti-oxidative stress roles via examples of NRF2 signaling (81), TNF-*α* signaling (82). On the other hand, studies have shown the neuroprotective effects of salvianolic acid A (Sal A), that has similar chemical properties with Sal B, in a photoreceptor degenerative model (83). A marked limitation of applying salvianolic acids in clinical assessments is their low stability in buffers with plasma pH (84).
3. Tanshinone IIA (Tan IIA). Tan IIA is another compound found in the root of Danshen, and belongs to a group of diterpenes called tanshinones. Tan IIA and its derivative sodium tanshinone IIA sulfonate carry o-naphthoquinone chromophore and provide anti-oxidation protection to retinal (85) and RPE cells (86) in stress-related models. A notable challenge of applying Tan IIA in clinical assessments is the poor oral bioavailability and water solubility (87), even though sodium tanshinone IIA sulfonate has improved water-soluble property (88). Sal B and Tan IIA have not been largely applied to IRD models and patients. Promisingly, the extracts from *salvia miltiorrhiza* bunge (containing Sal B and Tan IIA) could improve retinal morphology and function in *rd10* mice via the inhibition of oxidative stress by regulating the NRF2/HO-1 pathways (89).
4. Rutin. Rutin is also known as rutoside, a flavonoid that can be extracted in several plants, including tea leaves, citrus fruits, buckwheat, asparagus, and apples (90). Rutin inhibited cataractogenesis by maintaining the activity of antioxidant proteins (91, 92), and also delayed the photoreceptor degeneration in streptozotocin-induced diabetic retinas by directly regulating anti-apoptotic and antioxidant pathways (93, 94). In addition, *ginkgo biloba* extracts, procyanidin B2 and rutin, promoted RPE cell survival against t-BHP-induced apoptosis, suggesting their therapeutic potentials in treating age-related macular degeneration (AMD) (95). However, rutin has not been largely practiced in IRD-related trials.
5. Quercetin. Quercetin is a flavonoid found in fruits and vegetables, such as peppers, onions, berries, broccoli and red apples (96, 97), and its extraction is relatively easy (98, 99). The compound has two pharmacodynamic groups: a catechol group in the B ring and a 3-position OH group. Quercetin upregulated antioxidant peroxiredoxins through activation of the pro-survival signaling such as NRF2 and HO-1 signaling in models of AMD (100–102) and diabetic retinopathy (103, 104), and promoted the photoreceptor survival in NaIO_3_-treated mice (105). Moreover, quercetin downregulated photo-oxidative stress in light-damage photoreceptors by inhibition of the heterodimer binding of c-Jun and c-Fos proteins involved in the AP-1 pathway (106). More importantly, quercetin promoted the cone survival and functions in *rd10* mice during the period of persistent rod degeneration by reducing the expression of oxidative stress markers (107). Since its effective antioxidant and anti-inflammatory properties have been well observed in treating ocular diseases (108–110), quercetin can be a good candidate for second-line therapy to RP treatment.
6. Lutein. Lutein is a dietary carotenoid, found in various plants, particularly in green leafy vegetables such as spinach, kale, and broccoli. Its extraction from plants is not complicated (111–113). The neuroprotective effects of lutein in treating ocular diseases have been well documented (114–116), including its profound therapeutics in AMD treatment (117–119). In addition, 1-week treatment of lutein rescued rods and cones in *rd10* mice and reduced the reactive gliosis of Müller cells and inflammatory response (120). A 24-week lutein supplementation significantly preserved the visual field in placebo-controlled clinical trial on RP patients (121), however, inconsistent findings were obtained in other studies (122, 123).
7. Safranal. Safranal is a component extracted from stigmas of saffron (*Crocus sativus* L.) (124). Saffron extracts including safranal improved anti-inflammation and retinal functions in glaucoma models (125) and POAG trials (126, 127), and preserved photoreceptor and RPE cell survival in AMD models (128–130) and trials (131–133). In addition, the dietary supplementation of safranal prolonged photoreceptor survival, ameliorated the loss of retinal function, and improved the vascular network in *Rho^P23H/P23H^* rats (134). The neuroprotection to rod photoreceptor by safranal can be further exemplified in light-damage models (135, 136). Therefore, safranal or saffron extracts may have the promising therapeutic potential in RP treatment. However, the application of saffron extracts exhibits dose-dependent adverse effects (137). Hence, the clinical and experimental studies with safranal or saffron extracts usually adopted dosages of milligrams, showing minimum adverse effects.
8. Curcumin. Curcumin is the active compound extracted from turmeric ginger (*Zingiber officinale* Roscoe) (138, 139). Curcumin is a powerful antioxidant and anti-inflammatory agent that has been used in traditional Chinese medicine and widely used in clinical applications (140). In particular, curcumin treatment upregulated the expression of rod- and cone-specific genes and translocated rhodopsin to rod outer segment in *Rho^P23H/P23H^* rats. Curcumin treatment also reduced the endoplasmic reticulum stress in retinas (141). A similar rescue result by curcumin was reported in *P23H* swine model (142). Neuroprotective effects of curcumin on the photoreceptor survival can be seen in N-methyl-N-nitrosourea (MNU)-treated rats (143). However, clinical studies have shown adverse effects at high doses (>12 g/daily) of curcumin (144).

## 7 Treatment of retinal neurodegenerative diseases requires multidisciplinary collaboration

Genes, proteins, and lipids in the photoreceptor cells of retinitis pigmentosa animal models were highly oxidized, and oxidative damage was present in retinitis pigmentosa regardless of genotype (145). Retinitis pigmentosa is a disease caused by multiple genetic factors, and the path of disease expansion and rate of degeneration vary from person to person. Treatments that target different genes mutations are expensive and not efficient. The ideal solution would be to develop a treatment that can treat retinitis pigmentosa caused by all the different genetic defects. At the same time, treatments of early stage of degeneration of retinitis pigmentosa caused by genetic defects in rod photoreceptor cells will be required. Oxidative damage is related to the pathophysiology of retinitis pigmentosa such as death of rods and cones, and retinal inflammation may become a common therapeutic target for retinitis pigmentosa. A limitation of current research is that plants for the use in retinitis pigmentosa have not yet been fully developed, and more plants with effective active ingredients are worth developing and applying in this field. Other botanicals, including Tibetan medicines (Saussurea medusa Maxim, known as “snow lotus”) (146–148) and health products (various types of tea and mulberry leaves) also have antioxidant and neuroprotective effects. These herbal medicines can be developed for the treatment of retinal degenerative diseases (149, 150). In the future, we look forward to jointly developing new drugs for retinitis pigmentosa caused by oxidative damage through multidisciplinary collaboration with botanical scientists, pharmaceutical scientists, medical scientists, and ophthalmologists.

## 8 Future perspectives

In China, doctors currently prescribe several traditional Chinese medicine (TCM) to patients. The main principle of TCM is to restore the balance of yin and yang as well as the harmony of body and mind. Using the “look, smell, ask, and feel” method, doctors collect comprehensive information about the patient’s symptoms and signs. Based on this diagnosis, doctors select appropriate medicines and determine their dosages. A TCM prescription typically consists of multiple drugs, ranging from several to dozens. In TCM, it is believed that every medicine has a 70% therapeutic effect and a 30% potential for side effects. Furthermore, a famous ancient book from the Former Han Dynasty called “Huangdi Neijing” categorizes Chinese medicine into four groups based on toxicity: highly toxic, moderately toxic, mildly toxic, and non-toxic. Medicines can not only cure diseases but also cause them or have lethal effects. As a result, ensuring the safety of TCM prescriptions is paramount. The Pharmacopeia and literature have clear warnings regarding the cautious use or avoidance of toxic medicines.

While some patients travel to Western countries seeking gene therapy, others, due to genetic incompatibility or economic factors, opt for more affordable TCM treatments upon returning to China. Encouragingly, many have experienced positive therapeutic effects. Western medicine primarily targets specific or multiple disease-related factors, while TCM aims to rebalance the overall yin and yang in the body for therapeutic outcomes. Herbal medicine is often considered an alternative or complementary treatment option (151).

Research-based repositories of natural products are available, for example, the National Cancer Institute Natural Products Repository of NIH offers thousands of plant samples and resulting extracts, including Traditional Chinese Medicinal Plant Extracts Library, for drug screening studies. The eight above-mentioned compounds and associated herbs can be found in this repository, although a large majority of herbal extracts have not been tested in the treatment for inherited retinal diseases such as retinitis pigmentosa. In 2004, the FDA formulated the Botanical Drug Guidance, which is applicable to the clinical trials and inspection registration of new botanical drugs. Then the “Botanical Drug Development” guidance was announced in 2016, to address development considerations for late-stage trials and provide recommendations designed to facilitate botanical drug development (fda.org) (152). However, only some botanical New Drug Applications (NDAs) have been approved in the United States so far: Veregen in 2006, Fulyzaq in 2012 (152), Zoryve in 2023 (zoryve.com) and Filsuvez in 2023 (filsuvez.com). Scientists used this guidance to guide the research and development of botanical medicines (153). On the other hand, the European Medicines Agency (EMA) of the European Union proposed a draft “Guideline on quality of herbal medicinal products / traditional herbal medicinal products” in 2005 for the quality control of botanical medicines, then officially announced it in 2006, and a revised version (revision3) was released in 2022 (ema.europa.eu). Although Chinese pharmacy and Western pharmacy are separate disciplines in China, some scholars argue that the two can complement each other and have synergistic effects (154–156), thus surpassing the efficacy of either approach alone. In addition, we found in clinical practice that when doctors are seeing patients in outpatient clinics, genetic testing department and clinical trial staff are participating at the same time. In the future, the treatment of patients with retinitis pigmentosa can be combined with personalized medicine (157, 158) or comprehensive medical care. For example, the genotype of patients with retinitis pigmentosa can be detected first, then genetic testing can more accurately classify/ diagnose inherited retinal diseases and relevant drug treatment can be formulated based on the genotype.

TCM was introduced to Japan and, after over a thousand years of adaptation, has evolved into Kampo medicine, tailored to the Japanese constitution. In the future, TCM may be adjusted to suit the constitution of people from different regions and become a form of medicine applicable to individuals worldwide. Given the diverse medicinal plants found across the globe due to variations in soil, climate, and region, it is crucial to fully explore and develop medicinal herbs derived from these plants. Additionally, developing new drugs from the organic compounds present in these herbal extracts, combining them with gene therapy, cell therapy, and other innovative approaches, holds great value in overcoming rare human diseases and improving physical and mental well-being.

## Data Availability

The original contributions presented in the study are included in the article/supplementary material, further inquiries can be directed to the corresponding author.
